# Reducing calorie sales from supermarkets – ‘silent’ reformulation of retailer-brand food products

**DOI:** 10.1186/s12966-017-0559-y

**Published:** 2017-08-23

**Authors:** Jørgen Dejgård Jensen, Iben Sommer

**Affiliations:** 1 University of Copenhagen, Department of Food and Resource Economics, Rolighedsvej 25, 1958 Frederiksberg C, Denmark; 2 Danish Cancer Society, Strandboulevarden 49, 4100 København Ø, Copenhagen, Denmark

**Keywords:** Health promotion, Food product reformulation, Consumer demand

## Abstract

**Background:**

Food product reformulation is seen as one among several tools to promote healthier eating. Reformulating the recipe for a processed food, e.g. reducing the fat, sugar or salt content of the foods, or increasing the content of whole-grains, can help the consumers to pursue a healthier life style. In this study, we evaluate the effects on calorie sales of a ‘silent’ reformulation strategy, where a retail chain’s private-label brands are reformulated to a lower energy density without making specific claims on the product.

**Methods:**

Using an ecological study design, we analyse 52 weeks’ sales data – enriched with data on products’ energy density - from a Danish retail chain. Sales of eight product categories were studied. Within each of these categories, specific products had been reformulated during the 52 weeks data period. Using econometric methods, we decompose the changes in calorie turnover and sales value into direct and indirect effects of product reformulation.

**Results:**

For all considered products, the direct effect of product reformulation was a reduction in the sale of calories from the respective product categories - between 0.5 and 8.2%. In several cases, the reformulation led to indirect substitution effects that were counterproductive with regard to reducing calorie turnover. However, except in two insignificant cases, these indirect substitution effects were dominated by the direct effect of the reformulation, leading to net reductions in calorie sales between −3.1 and 7.5%. For all considered product reformulations, the reformulation had either positive, zero or very moderate negative effects on the sales value of the product category to which the reformulated product belonged.

**Conclusions:**

Based on these findings, ‘silent’ reformulation of retailer’s private brands towards lower energy density seems to contribute to lowering the calorie intake in the population (although to a moderate extent) with moderate losses in retailer’s sales revenues.

## Background

Food product reformulation is seen as one among several tools to promote healthier eating [1–3]. By reformulating the recipe for a processed food, e.g. reducing the fat, sugar or salt content of the foods, or increasing the content of whole-grains, food manufacturers can help consumers pursue a healthier life style, even without changing their dietary behavior.

The research literature on food product reformulation aiming at health improvements has considered the size of the potentials for health promotion via product reformulation, the industry’s economic incentives to reformulate, or the possible barriers for such reformulation to occur. Regarding health promotion potentials, a number of studies have considered reduction of salt content [4, 5], replacement of trans-fatty acids (TFA) [6, 7] in food products, or enhancement of whole-grain from foods [8]. Often, such studies use a static approach ignoring any behavioural adjustments to the reformulation among the consumers, and thus find that such product reformulations are promoting public health.

Studies addressing food producers’ incentives to undertake product reformulation tend to fall in one of two categories [9]: supply-side incentives to reformulate driven by costs of ingredients - which in turn may be driven by e.g. agricultural policy - and demand-side incentives driven by the possibility to reap competitive advantages from consumers’ demand for healthier products [10, 11]. Downs et al. [3] reviewed the literature on the effectiveness of policies to reduce the intake of TFA, such as bans or labelling. Regarding TFA in food, most of the reviewed studies found that policy interventions had led to reformulation of products to contain less TFA. The topic was also studied by Unnevehr & Jagmanaite [10] with a particular focus on stimulus to reformulate food products towards less content of TFA. Golan & Unnevehr [9] considered how food industry’s incentives to undertake product reformulation may be affected by agricultural policy affecting food manufacturers’ input costs. Marotta et al. [11] examined the role of product reformulation for the food industry and whether this strategy can help companies to gain a competitive advantage. They found that improved consumer information is necessary in order for such mechanisms to be effective.

As an alternative to promote reformulated products via information about their healthiness, another product reformulation strategy is to make it ‘silent’, i.e. not to announce the reformulation explicitly to the consumers (except on standard nutrition labels). This strategy does not have the potential competitive advantage of appealing to health-oriented consumers for the individual product. Instead, the manufacturer’s or retailer’s motivation to undertake such silent reformulation could be to enhance the health profile of its corporate brand, considering the reformulation as a part of its Corporate Social Responsibility (CSR) strategy. This type of reformulation strategy seems to have attracted rather little interest in the research literature, even though considerable research interest has been devoted to the potential roles of food industry self-regulation in halting the worldwide increasing trend in overweight, obesity and co-morbidities [12].

The research literature has also devoted relatively little attention to the consumers’ adaptation to reformulation of food products and hence its effectiveness in reducing the consumers’ intake of calories. Food reformulation towards lower fat, sugar or salt content might induce compensation effects – that the consumers perceive a need (or allowance) to compensate for the reduced contents of such substances by increasing their consumption of other products with these substances. Product reformulation might be expected to change the perceived quality of the products - and hence to affect the comparison between the reformulated product and its substitutes, for example because the reformulated product is considered to be ‘unnatural’ or a ‘diet’ product. To the extent that consumers respond to such perceived quality changes, these responses may enhance or undermine the effectiveness.

In the present analysis, consumers are assumed to make their consumption decisions regarding a product, based on their perception of the product’s attributes (taste, healthiness, price,...) relative to those in potential substitute products within the same product category, but probably with slightly different characteristics. If a reformulation enhances the perception of one attribute without deteriorating other attributes, the reformulation would be expected to induce an increase in the consumption of that product. On the other hand, if the reformulation leads to a perceived improvement in some attributes but a perceived negative effect on other attributes, the sign of the demand response needs to be based on empirical analysis. It is hypothesized that such substitution effects influence the impact of the product reformulation on consumers’ calorie consumption, as well as the retailer’s sales value. The key research questions are, whether such substitution effects are significant and whether they are supportive of public health goals and of industry’s incentive to undertake such reformulation.

We evaluate the effects of a ‘silent’ reformulation strategy, namely to reformulate a retail chain’s private-label brands to a lower energy density without announcing the reformulation to the consumers. Such a strategy has been exercised by one of the major retail chains in Denmark. The modest changes in the food composition pursuant of the reformulation only had impact on the nutrient content, no impact on price, whilst no or insignificant changes in the sensory quality of the products. Nutrition fact labels on the products were updated as a consequence of the reformulation. If consumers have not noticed changes on the nutrition facts label nor in sensory characteristics, their product choices are hypothesized to be unaffected by the reformulation. On the other hand, if the consumers perceive a change in sensory characteristics or have noticed a changed in nutritional characteristics, their choices could be affected. The direction of such changes is however indeterminate, a priori.

We focus on the consumers’ adaptation to such reformulation in eight food product categories in terms of possible changes in their composition of purchases within these categories, based on sales data from a Danish retail chain.

## Methods

### Study design and data

In relation to an epidemiological terminology, the study design can be characterised as an ecological design [13], where the development over time in aggregate sales of individual products is seeked explained by the development in relevant explanatory variables, such as prices and timing of product reformulation.

The dataset for the analysis consists of detailed weekly sales data from one of the larger food retail chains in Denmark for a 52-week period, from the beginning of March 2013 till the end of February 2014. The sales data have been enriched by information about energy content in all products (kcal/100 g or kcal/100 ml).

Data concerning product reformulation for eight products were also supplied by the retail chain, including the date (week number), the character of the reformulation and its effect on the product’s energy content.

### Theoretical assumptions and functional form

For the empirical analysis of each product category, we have chosen the linearized Almost Ideal Demand (AID) functional specification [14]. The AID model implies that commodity *i*‘s share *w*_*i*_ of the product category sales value can be specified as a linear function of the logarithmic prices *p* and the real budget for the product category *y*/*P* (where *P* is the composite price index of all prices in the product category, calculated according to the Stone price index formula, ln*P* = ∑_*i*_ *w*_*it*_ ⋅ ln *p*_*it*_).

In order to investigate the potential effects of product reformulation, we have augmented the AID model with a dummy variable *D*, which assumes the value zero before the reformulation, and the value one after the reformulation, hence

$$ {w}_{it}={\alpha}_i+{\sum}_j{\alpha}_{ij}\cdot \ln {p}_{jt}+{\beta}_{iy}\cdot \ln \left({y}_t/{P}_t\right)+{\gamma}_i\cdot {D}_t+{\varepsilon}_{it},i=\left\{1,\dots, n\right\} $$

A linear trend variable and a dummy variable representing the Christmas season (all weeks in December), were included to account for trend and seasonality effects (although the products in the study are not suspected to be subject to strong seasonal variation). Furthermore, in order to adjust for first-order autocorrelation, an autoregressive AR (1) term was included in the estimation.

Adding-up yields ∑_*i*_ *α*_*i*_ = 1 , ∑_*i*_ *α*_*ij*_ = 0 , ∑_*i*_ *β*_*yi*_ = 0 , ∑_*i*_ *γ*_*i*_ = 0, given that the budget shares sum to one. At the individual consumer level, a theoretically consistent demand model should also fulfil properties of linear homogeneity and Slutsky symmetry. However, as the retailer sales data used in this study can be considered as an aggregate of several consumers, these assumptions cannot be expected to hold, and have therefore not been imposed on the econometric estimation.

Based on econometric estimation of the AID model, it is possible to separate out the effect of the reformulation dummy on the composition of product purchases at a given budget as *Δx*_*it*_|_*ΔD*_ = *γ* ⋅ *y*_*t*_/*p*_*it*_. This effect reflects possible indirect within-commodity-group substitution behaviour triggered by the reformulation. The substitution effect influences both the turnover of calories and the total sales value, within the respective product categories. Other effects, such as effects of price changes, overall sales volume changes or trend development could also be separated out, but this is outside the scope of this paper and is not presented.

In addition to the indirect substitution effects of the reformulation on calorie turnover, there is also a direct effect of product reformation in terms of changed energy density of the reformulated products, *Δk*_*i*_. Hence, total effect of the reformulation on calorie turnover can be determined as

$$ \varDelta K\left|{}_{reformulation}\right.={\sum}_i{x}_{it}\cdot \varDelta {k}_i+{\sum}_i{\left.{k}_i\cdot \varDelta {x}_{it}\right|}_{\varDelta D} $$

For sales value, the effect of the reformulation is given by:

$$ \varDelta R={\sum}_i{p}_i\cdot {\left.\varDelta {x}_{it}\right|}_{\varDelta D} $$

### Products

Within the considered data period, the retail chain reformulated nine of their private label food products with the aim to lower their energy content. The nine products and their reformulations are displayed in Table 1. Two of the product reformulations were implemented simultaneously in two close substitute products (orange-flavoured yoghurt and peach melba-flavoured yoghurt), and in the subsequent analysis, this double-reformulation is analysed as one combined setting.

**Table 1:** Overview of considered product reformulations and categories

Product	Change in energy density (%)	Date of reformulation	Category products	Reformulated product’s share of category’s calorie turnover	Reformulated product’s share of category’s sales value
	Kcal/ 100 g	Week/ year		Per cent	Per cent
Mayonnaise	720→600 (17)	36/2013	*Private-label mayonnaise,* Private-label light mayonnaise, Unbranded lemon mayonnaise, Brand 1 mayonnaise (tube), Brand 1 mayonnaise, Brand 1 lemon mayonnaise	51	38
Fruit yoghurt Orange Peach melba	93→77 (17) 90→74 (18)	14/2013	*Private-label peach melba yoghurt, Private-label orange yoghurt,* Banana/pear yoghurt, Pineapple/orange/mango yoghurt, Peach/passion fruit yoghurt, Berry-flavoured yoghurts, Low-calorie berry-flavoured yoghurts,	15 6	11 5
Pumpkin seed rye bread	240→232 (3)	22/2013	*Private-label pumpkin seed rye bread,* Private-label sunflower seed rye bread, Brand sunflower rye bread, Brand organic sunflower rye bread	14	12
Toasting bun	280→270 (4)	36/2013	*Private-label toasting bun,* Private-label whole-grain toasting buns, Brand toasting bun	64	60
Yoghurt bread	270→250 (7)	32/2013	*Private-label yoghurt bread,* Brand1 wholemilk bread, Brand1 yoghurt bread, Brand2 wholemilk bread, Brand 3 five-grain bread	43	38
Carrot buns	310→281 (9)	22/2013	*Private-label carrot buns,* Brand1 carrot buns, Brand1 other buns, Brand 2 buns	54	46
Whole-grain rolls	260→240 (8)	22/2013	*Private-label whole-grain rolls,* Brand1 multi-grain rolls, Brand2 Sandwich rolls, Brand 2 grain rolls, Brand 3 Whole-grain rolls	35	30
Chocolate muesli	410→400 (2)	18/2013	*Private-label chocolate muesli*, Private-label regular muesli, Brand1 chocolate cereals, Brand 2 chocolate cereals, Brand 3 chocolate cereals	21	21

Note: Week numbers represent the placement of the week in the calendar year. For instance, week 36 in 2013 spanned the dates September 2–8, 2013

As appears from the table, the extent of reformulation in terms of reduction in energy density has varied across the different products. For example, for mayonnaise and fruit-flavoured yoghurt, the calorie content was reduced by 16–17%, whereas for bread products the reduction was 5–10%, and for chocolate-flavoured muesli only 2–3%.

Most of the affected product categories can be considered as ‘high-frequency buys’, where the possible demand effects of a reformulation would be measureable within few weeks after the reformulation has been implemented. One exception may be mayonnaise, where households’ purchase frequency is likely to be lower and where it may take longer to detect an influence of the reformulation. However, as the data for mayonnaise spans more than a half year after the reformulation, we would still expect to be able to measure an effect, if any.

## Results

In the following, results are presented for the eight reformulations, based on econometric estimation of the demand model outlined in the previous section. In Table 2, the reformulations’ effects on calorie turnover and sales value are evaluated for the group of relatively close substitutes, which the product is presumed to belong to. Detailed econometric estimation results for each commodity category are given in Tables 3, 4, 5, 6, 7, 8, 9 and 10 in the Appendix. In general, the estimated models displayed satisfactory goodness-of-fit and only little sign of misspecification, such as heteroscedasticity, autocorrelation or non-normality in residuals.

**Table 2:** Direct and indirect effects of product reformulation on calorie turnover and sales value for product groups (per cent)

	- - - - - - - - - - - - - - Calories - - - - - - - - - - - - - - -			
	Direct reformulation	Reformulation-induced substitution	Total calorie	Sales revenue
Mayonnaise	−8.2%	0.7%	−7.5%	0.4%
Fruit yoghurt	−3.9%	0.1%^N.S.^	−3.8%	0.1% ^N.S.^
Rye bread with pumpkin seeds	−0.5%	0.7% ^N.S.^	0.2%	0.7% ^N.S.^
Toasting buns	−2.3%	−0.7%	−3.0%	−0.5%
Yoghurt bread	−3.2%	−0.5% ^N.S.^	−3.7%	−0.5% ^N.S.^
Carrot buns	−4.9%	0.7% ^N.S.^	−4.2%	0.4% ^N.S.^
Whole-grain rolls	−2.7%	0.0%	−2.7%	−0.1%
Chocolate muesli	−0.5%	3.7% ^N.S.^	3.1%	2.2% ^N.S.^

N.S. Not significant

### Mayonnaise

The reformulation has led to a partial decrease in calories from the reformulated mayonnaise product by 17%, as indicated in Table 1, which contributes by about 8% reduction in the total calories from the group of mayonnaise products in this retail chain. The partial substitution effect of the reformulation increased the sale of the reformulated product by 2.6% (and hence the calories from this variety) at the cost of the private-label light variety, which decreased by 0.5% due to this substitution (the latter not statistically significant, though). An interpretation of this substitution effect is that consumers tend to prefer the taste of regular mayonnaise to that of light mayonnaise, but they are also concerned about the calories in the mayonnaise. When the calorie content of the regular variety comes closer to that of the light variety, their trade-off between the two varieties becomes more in favour of the regular variety, and they become more prone to choose this variety. In total, the reformulation has led to a 7.5% reduction in calorie sales in mayonnaise.

The reformulation-induced substitution effects on sales value are identical to those on calorie turnover for the individual products. However, because effects on turnover are weighted by products’ turnover shares, and effects on calories are weighted by products’ calorie shares, the total percentage reformulation-induced substitution effect differs for the two outcome measures. Hence, the increase in turnover due to the reformulation per se is relatively less than the corresponding contribution to change in calorie sales - but still positive. According to these results, the retailer thus did not lose revenue due to the reformulation of the private-label mayonnaise.

### Fruit yoghurt

The two reformulated yoghurts represent 11–15 and 5–6% of total fruit yoghurt sales, respectively, and neither of them could be considered as dominating in terms of sales. The direct reformulation effect constituted around 17% reduction in calorie content for both reformulated product types. Taking into account that these two products represent around 20% of total calorie sales, this direct effect amounts to 3.9% of total calorie turnover from the fruit yoghurts. The reformulation-induced substitution effects were not statistically significant, and the results in Table 2 also indicates very small contributions to the change in calorie turnover and sales value from this effect.

### Rye bread with pumpkin seeds

One private-label variety of rye bread with pumpkin seeds was reformulated to contain fewer sunflower seeds and more pumpkin seeds and wholegrain rye, thus reducing the calories from 240 to 232 per 100 g. For the rye bread reformulation, the direct reformulation effect led to a 3.3% reduction in the reformulated bread, which implied a 0.5% partial reduction in the total calories from this product category. Reformulation-induced substitution effects led to a (not statistically significant) 0.7% increase in the calorie turnover - and in sales revenue. The net effect of the reformulation on calorie turnover was an insignificant 0.2 (=0.7–0.5) per cent increase, and the partial effect of the reformulation on sales value was an (insignificant) increase of 0.7%. Hence, the reduction in calorie turnover due to the reformulation could be done without reducing the sales value of this group of rye bread.

### Toasting buns

One private-label ‘normal’ toasting bun (“krydderboller” in Danish) was reformulated to contain less fat and sugar, thus reducing the calorie content from 280 to 270 kcal per 100 g. The direct effect of the reformulation was a reduction of 3.6% calorie content in the reformulated product – which impacted the average calorie turnover in toasting buns by 2.3%. Reformulation-induced substitution effects implied a (weakly significant) shift from the reformulated private-label product to the whole-grain private-label variety, implying a net reduction in calorie turnover of 0.7%. Hence, the reformulation implied a 3% reduction in calorie turnover from toasting buns. The substitution effect also implied a reduction in sales revenue of 0.5% from these buns.

### Yoghurt bread

In the category of light bread, the retailer’s private label yoghurt bread was reformulated to contain more rye flour and less fat, which reduced the calorie content from 270 to 250 kcal/100 g. The direct effect of the reformulation was a 7.4% reduction in the calorie content of the reformulated product, and thus to a 3.2% reduction for the product category as a whole. Substitution effects induced by the reformulation led to (insignificant) decrease in the sale of the reformulated product and slight increases in some of the other products, leading to an overall 0.5% reduction in calorie sales, and a corresponding effect on sales value.

### Buns with carrot

Private-label buns with carrot were reformulated to contain less sunflower seeds, more durum seeds, more carrot and no eggs. The direct effect of the reformulation was a reduction of the calorie content in these buns with 9.4% – and as these buns constitute about half of the considered product category, this implied a direct effect on the calorie turnover of this product category by 4.9%. Although statistically insignificant, the reformulation-induced substitution effects led to an increased sale of the reformulated buns, at the cost of another variety, and the net effect of this substitution was an increase of 0.7% in calorie turnover. Hence, the reformulation yielded a 4.2% reduction in calorie turnover, and a 0.4% increase in sales value of the buns within this product category.

### Whole grain breakfast rolls

Private-label whole-grain rolls were reformulated to contain less oil, which reduced the energy density from 260 to 240 kcal/100 g. It may not be completely evident, which products are likely to be close substitutes to these rolls – and especially if this would be other types of rolls for lunch sandwiches, or other types of rolls on the breakfast table. In the case of breakfast use, the sales data contain very little substitute products within the fresh bread category, and hence the product reformulation could be expected to induce relatively little substitution in the sales, and the direct reformulation effect (i.e. a 7.7% reduction in calorie turnover from the category) would be the dominating effect.

On the other hand, if other types of sandwich rolls could substitute the reformulated rolls, we consider six products as close substitutes. The direct effect of the reformulation on the reformulated product was an 8% reduction in the calorie content. As the reformulated product represented around one third of the total sales within this category, this implies a reduction of 2.7% in the product category. Reformulation-induced substitution effects implied some shifts in the composition of this product category – however with a zero effect on total calorie turnover. Although this substitution effect was relatively small on the reformulated product, it seems to have induced a shift between some of the other product varieties. The reformulation-induced substitution effects implied a 0.1% reduction in the sales value from this product category. These somewhat peculiar substitution effects may have to do with the above considerations about which products are the actual substitutes for the reformulated whole grain rolls.

### Chocolate muesli

The retailer has reformulated its private-label chocolate muesli product to contain more grains (wheat and oats) and less salt, and reduce the energy density from 410 to 400 kcal/100 g. The direct effect of the reformulation was a 2.4% reduction in calorie content of the reformulated chocolate muesli. As this product constituted around 20% of the product category, this meant a reduction in the calories from this category of 0.5%. The substitution effects of the reformulation implied an (insignificant) increase in the sales of the private-label regular muesli products, at the cost of especially one of the other brands. This substitution effect led to a 3.7% increase in calorie sales, from the product category, and hence a net increase of 3.1% in calorie turnover. The reformulation-induced substitution effect on sales value was an increase of 2.2%.

## Discussion

For all products, the direct effect of product reformulation was a reduction in the sale of calories from the respective product categories. In several of the cases, the reformulation led to indirect substitution effects that to some extent were counterproductive with regard to reducing calorie turnover – but in all cases except for chocolate muesli and rye-bread, these indirect substitution effects were dominated by the direct effect of the reformulation. For all considered product reformulations, except that of toasting buns, the reformulation had either positive, zero or very moderate negative effects on the sales value of the product category to which the reformulated product belonged. Consequently, the examples suggest that silent product reformulation can be a somewhat effective and economically feasible strategy for retailers to reduce their customers’ consumption of calories from their products.

It is worth noting that in most of the considered cases, the modelled reformulation did not have statistically significant effects on the composition of consumption within the respective product categories. Hence, ‘silent’ product reformulation does not seem to scare consumers away, nor attract new consumers. Mayonnaise is an exception from this general pattern, as the reformulation seems to have increased the demand for the reformulated private-label product, mainly at the cost of the ‘light’ private-label product.

Buttriss [14] pointed at some of the challenges that may have to be met in relation to food product reformulation to a lower fat or sugar content. One challenge is the constraints of food legislation for some sectors, such as minimum requirements to the fat content of e.g. a chocolate product to be marketed as such, or regulation of nutrition and health claims, which may constrain producers’ publicising changes in fat content. In addition, the Danish legislation on food labelling requires that a food product to be labelled as ‘light’ or ‘low-fat’ should have an energy content of maximum 70% of the corresponding ‘regular’ product, which may also limit the scope and incentive for reformulation.

Another challenge is that manufacturers have an interest to retain the characteristics of the product that are attractive to consumers, making reformulation to reduce fat a costly and time-consuming exercise. Reducing sugar content also poses challenges because sugar’s role in foods is often more than simply providing sweetness, and different alternative approaches may be needed to provide the products with these attributes. Some of these alternative approaches have limitations, due to e.g. regulatory constraints, potential gastrointestinal consequences associated with some alternative ingredients, consumers’ resistance to foods containing many additives, or that the alternatives are often more costly than sugar.

The product reformulations considered in this study have been quite moderate, as also mentioned above. Previous studies [15–17] indeed suggest that gradual product reformations, with fairly small steps at the time and thus slowly familiarizing the consumers with changes in taste and other characteristics, can be a fruitful strategy for health-promoting product reformulations. Due to the rather moderate extent of the considered reformulations, the magnitude of the public health impact might also be limited. Nevertheless, the results of the analysis provide fairly clear indications of a reduction in calorie intake in the respective commodity groups.

### Limitations of the study

In the empirical analysis, we have adopted the assumption of a “representative consumer” purchasing the range of products in the considered retail chain, and we have estimated behavioural parameters reflecting utility maximization behaviour of this representative consumer. This assumption implies that the composition of consumers – and hence the average preferences - is assumed to be stable throughout the 52-weeks data period, or develops along a linear trend.

Furthermore, we have assumed weak separability in consumption in the sense that the composition of specific products within a certain (narrow) product category (e.g. the mayonnaise category or chocolate-flavoured breakfast cereals category) is independent of the relative prices of products outside this product category [18]. This assumption has enabled us to estimate separate systems of demand equations within each product category. But the separability structure also imposes restrictions on the substitution patterns that can be detected by the analysis. In the analysis, we have considered substitution effects between the reformulated product and its close substitutes. In most cases, the range of close substitutes was relatively straightforward. However, for some of the bread products, some subjective judgement as to which products were ‘close’ substitutes, and which were ‘less close’ substitutes, was necessary. Although the delimitation of these product categories was done to the best of the authors’ capabilities, this may induce some uncertainty to the results for these products.

The product reformulation was represented as a ‘dummy’ (or binary) variable in the econometric analysis. This approach implies the assumption that from the date of reformulation, the consumers can only buy the reformulated version of the product - and not the ‘old’ version. For products with a shelf life of about 1 week (such as fruit yoghurt or fresh bread), this is not an unrealistic assumption. But for chocolate muesli and mayonnaise, there may have been a longer transition period, where the individual stores have emptied their shelves with the ‘old’ version, before they introduced the reformulated version of the product, and this may have introduced uncertainty to the results for these two products.

The ‘dummy’ variable representation also assumes that the consumers either consider the reformulation in their purchase decision from day one - or do not consider it at all. Again, this may introduce some uncertainty to the results.

The econometric estimation assumes stability in the population of consumers and its composition - or as a minimum that any development in this composition can be captured by the linear trend variable included in the estimation equations. The considered retail chain has however expanded quite significantly during the 52 weeks of consideration. For this reason, this assumption could be questioned - especially if new stores have been established in areas with ‘new’ socio-demographic characteristics, compared to those of the ‘old’ stores.

The analysis is based on sales data from only this one retail chain. Thus, interpreting the results as representative for the population of consumers has to be based on an assumption that their choice of store has not been affected by the product reformulation. It might be hypothesized that changes (e.g. assortment changes, price changes, product reformulations) could induce substitution effects in the consumers’ choice of store. Address of such effect across stores is however very difficult and has not been possible in this study. It might be presumed that such substitution effects would undermine the possible health promoting effects, because consumers seek ‘un-reformulated’ products in other stores. On the other hand, the reformulation may also attract new customers. However, as the considered reformulations are generally quite moderate, and because the chain’s stores already have fairly broad assortments of relevant substitutes, such across-store substitution effects are considered to be negligible. Although this may appear a realistic assumption, it has not been verified in this study, and this may also add to the uncertainty of the results.

## Conclusion

This study has evaluated the effects of ‘silent’ reformulating a retail chain’s private-label brands to a lower energy density, based on weekly sales data for a period of 52 weeks from a Danish retail chain. The analysis distinguishes between the direct effect of the lower energy density in the reformulated product and the indirect effects due to reformulation-induced product substitution. For seven out of the eight reformulated products considered in the study, the reformulation led to a reduction in calorie sales (defined as the sum of the direct and indirect effects from the reformulation). For chocolate muesli, a counter-productive (but statistically insignificant) substitution effect outweighed the direct effect of the reformulation. In most cases, the estimated reformulation-induced substitution effects were small and statistically insignificant. Hence, the sales value was only affected to a little extent - and only negatively affected in two of the eight cases considered.

Based on these findings, ‘silent’ reformulation of retailer’s private brands towards lower energy density, seems to be a promising strategy for the retail sector to contribute to lower calorie intake in the population, and thus supportive of public health goals and industry’s incentive to undertake also modest and silent reformulations.

## Appendix

**Table 3:** Detailed results for mayonnaise

	Brand 1, tube	Private label light	*Private label*	Unbranded lemon	Brand 1, lemon
Initial share of calories	6.2%	12.2%	50.9%	0.3%	1.9%
Initial share of sales value	13.7%	16.1%	37.6%	0.5%	1.8%
*AID model estimation results*					
Ln P_Brand 1, tube_	0.1937	0.1072	0.5578	−0.0047	−0.0776
	(0.1669)	(0.2123)	(0.3727)	(0.0430)	(0.1428)
Ln P_Private label light_	−0.1631	−0.3367	−0.5005	−0.0184	−0.1574
	(0.2208)	(0.3015)	(0.5126)	(0.0660)	(0.2053)
Ln P_Private label_	−0.0469	−0.0145	0.3002	0.0288	−0.0221
	(0.0529)	(0.0795)	(0.1248)	(0.0175)	(0.0536)
Ln P_Unbranded lemon_	0.0009	−0.0060	−0.0398	0.0094	0.0056
	(0.0168)	(0.0200)	(0.0361)	(0.0038)	(0.0125)
Ln P_Brand 1_	0.0600	0.0515	0.1887	0.0026	−0.0214
	(0.0063)	(0.0064)	(0.0128)	(0.0013)	(0.0040)
Ln P_Brand 1 lemon_	0.0043	−0.0012	0.0187	−0.0001	0.0066
	(0.0124)	(0.0152)	(0.0274)	(0.0031)	(0.0098)
Real budget	0.0111	−0.0139	−0.0204	0.0013	0.0027
	(0.0056)	(0.0062)	(0.0117)	(0.0012)	(0.0039)
Reformulation dummy	0.0107	−0.0045	0.0260	−0.0009	−0.0094
	(0.0040)	(0.0067)	(0.0098)	(0.0016)	(0.0047)
a10	0.1123	0.0207	0.1091	0.0058	−0.0227
	(0.0412)	(0.0513)	(0.0913)	(0.0103)	(0.0343)
Trend	−0.0005	−0.0004	0.0000	0.0000	−0.0001
	(0.0003)	(0.0005)	(0.0007)	(0.0001)	(0.0003)
Christmas dummy	−0.0025	−0.0021	−0.0117	0.0007	0.0093
	(0.0049)	(0.0070)	(0.0115)	(0.0015)	(0.0047)
AR (1)	−0.0083	0.5079	0.2058	0.7861	0.6214
	(0.1816)	(0.1553)	(0.1785)	(0.1212)	(0.1336)
Intercept	−0.1459	0.6999	−0.6859	−0.0436	0.5860
	(0.2842)	(0.4293)	(0.6790)	(0.1042)	(0.2956)
Root MSE	0.0056	0.0067	0.0121	0.0014	0.0043
R^2^	0.90	0.85	0.94	0.84	0.83
R^2^-adj	0.86	0.80	0.92	0.78	0.78

**Table 4:** Detailed results, fruit yoghurt

	Private label peach melba	Private label orange	Pear/ banana	Pineapple/ orange/ mango Light	Berry-flavoured
Initial share of calories	15.4%	5.8%	44.9%	4.0%	21.4%
Initial share of sales value	11.4%	5.3%	37.1%	8.9%	20.3%
AID model estimation results					
Ln P_Private label peach melba_	0.000006	0.000002	−0.000004	−0.000001	−0.000002
	(0.000001)	(0.000000)	(0.000001)	(0.000001)	(0.000001)
Ln P_Private label orange_	0.000001	0.000002	0.000000	0.000001	−0.000004
	(0.000001)	(0.000001)	(0.000001)	(0.000001)	(0.000002)
Ln P_Pear/banana_	0.000005	0.000006	0.000004	−0.000006	0.000002
	(0.000002)	(0.000001)	(0.000003)	(0.000002)	(0.000003)
Ln P_Pineapple/orange/mango Light_	0.000001	0.000001	−0.000005	0.000015	−0.000002
	(0.000001)	(0.000001)	(0.000001)	(0.000001)	(0.000001)
Ln P_Berry_	−0.000001	0.000001	−0.000006	−0.000001	0.000021
	(0.000002)	(0.000001)	(0.000002)	(0.000003)	(0.000003)
Ln P_Berry Light_	−0.000002	0.000000	−0.000010	−0.000002	−0.000006
	(0.000001)	(0.000001)	(0.000001)	(0.000001)	(0.000002)
Real budget	0.000011	0.000013	−0.000010	−0.000005	0.000009
	(0.000004)	(0.000003)	(0.000005)	(0.000004)	(0.000005)
Reformulation dummy	−0.004530	0.000008	0.002339	−0.000570	0.005419
	(0.003120)	(0.002320)	(0.004070)	(0.006260)	(0.008760)
Trend	−0.000150	0.000004	−0.000130	−0.000920	0.001534
	(0.000056)	(0.000041)	(0.000073)	(0.000577)	(0.000626)
Christmas dummy	−0.001470	0.000176	−0.006100	−0.000950	0.005525
	(0.002460)	(0.001760)	(0.003190)	(0.004400)	(0.006080)
AR (1)	−0.402090	−0.186420	−0.263600	0.879930	0.862313
	(0.147000)	(0.181400)	(0.174500)	(0.093100)	(0.080600)
Intercept	0.095738	0.048126	0.362423	0.113309	0.161103
	(0.006660)	(0.004570)	(0.008380)	(0.023100)	(0.026200)
Root MSE	0.0044	0.0028	0.0053	0.0055	0.0076
R^2^	0.91	0.83	0.96	0.95	0.93
R^2^-adj	0.89	0.78	0.95	0.93	0.91

**Table 5:** Detailed results - rye bread with pumpkin seeds

	*Private label pumpkin*	Private label sunflower	Brand organic sunflower
Initial share of calories	13.8%	12.0%	0.0%
Initial share of sales value	11.8%	10.4%	0.0%
AID model estimation results			
lnP_Private label pumpkin_	0.0000	0.0000	0.0000
	(0.0000)	(0.0000)	(0.0000)
lnP_Private label sunflower_	0.0000	0.0000	0.0000
	(0.0000)	(0.0000)	(0.0000)
lnP_Brand organic sunflower_	0.0000	0.0000	0.0000
	(0.0000)	(0.0000)	(0.0000)
lnP_Brand other sunflower_	0.0000	0.0000	0.0000
	(0.0000)	(0.0000)	(0.0000)
Real budget	0.0000	0.0000	0.0000
	(0.0000)	(0.0000)	(0.0000)
Reformulation dummy	−0.0041	−0.0048	−0.0015
	(0.0054)	(0.0049)	(0.0034)
Trend	−0.0002	−0.0002	0.0001
	(0.0002)	(0.0002)	(0.0001)
Christmas dummy	−0.0003	0.0001	0.0006
	(0.0033)	(0.0031)	(0.0021)
AR (1)	−0.1108	−0.0573	−0.3948
	(0.2253)	(0.2215)	(0.2373)
Intercept	0.0942	0.0894	0.0251
	(0.0075)	(0.0068)	(0.0051)
Root MSE	0.0057	0.0051	0.0041
R^2^	0.99	0.99	0.97
R^2^-adj	0.99	0.99	0.96

**Table 6:** Detailed results – Toasting buns

	Private-label whole grain	*Private-label*
Initial share of calories	35.6%	0.1%
Initial share of sales value	39.9%	0.1%
*AID model estimation results*		
Ln P_Private-label whole grain_	−0.2273	0.2328
	(0.0501)	(0.0514)
Ln P_Brand_	0.0008	−0.0013
	(0.0043)	(0.0044)
Ln P_Private-label_	0.0345	0.0110
	(0.1968)	(0.2015)
Real budget	0.1398	−0.1372
	(0.0185)	(0.0189)
Reformulation dummy	0.0245	−0.0246
	(0.0127)	(0.0130)
Trend	0.0006	−0.0006
	(0.0004)	(0.0004)
Christmas dummy	−0.0284	0.0285
	(0.0144)	(0.0147)
AR (1)	−0.1913	−0.1923
	(0.1700)	(0.1699)
Intercept	−0.6575	1.5197
	(0.5805)	(0.5942)
Root MSE	0.0256	0.0262
R^2^	0.83	0.83
R^2^-adj	0.80	0.79

**Table 7:** Detailed results - yoghurt bread

	Brand 2 Wholemilk bread	Brand 1 Wholemilk bread	*Private label yoghurt bread*	Brand 1 Yoghurt bread
Initial share of calories	0.0%	30.2%	43.2%	0.0%
Initial share of sales value	0.0%	31.8%	38.2%	0.0%
*AID model estimation results*				
lnP_Brand 2 Wholemilk bread_	0.1122	−0.0442	−0.0484	0.0000
	(0.0330)	(0.0172)	(0.0182)	(0.0001)
Ln P_Brand 1 Wholemilk bread_	0.2815	−0.6210	0.2239	0.0001
	(0.0825)	(0.0430)	(0.0456)	(0.0003)
Ln P_Private label yoghurt bread_	0.2191	0.5111	−1.1483	0.0005
	(0.3671)	(0.1913)	(0.2028)	(0.0015)
Ln P_Brand 1 Yoghurt bread_	−0.0251	0.0094	0.0058	0.0001
	(0.0181)	(0.0094)	(0.0100)	(0.0001)
Ln P_Brand 3, 5-grain bread_	0.0612	0.1295	0.1951	0.0002
	(0.0574)	(0.0299)	(0.0317)	(0.0002)
Real budget	0.2060	−0.0789	−0.0597	−0.0002
	(0.0488)	(0.0254)	(0.0270)	(0.0002)
Reformulation dummy	−0.0013	0.0085	−0.0100	0.0000
	(0.0352)	(0.0183)	(0.0194)	(0.0001)
Trend	0.0006	−0.0005	−0.0001	0.0000
	(0.0013)	(0.0007)	(0.0007)	(0.0000)
Christmas dummy	0.0085	−0.0166	−0.0001	0.0002
	(0.0501)	(0.0261)	(0.0277)	(0.0002)
Intercept	−3.3966	1.2240	2.3714	−0.0002
	(1.2671)	(0.6603)	(0.7001)	(0.0053)
Root MSE	0.0603	0.0314	0.0333	0.0003
R^2^	0.62	0.93	0.88	0.28
R^2^-adj	0.53	0.92	0.85	0.11

**Table 8:** Detailed results - rolls with carrot

	*Private label carrot rolls*	Brand 1 carrot rolls	Brand 1 whole-grain rolls
Initial share of calories	53.7%	0.0%	28.2%
Initial share of sales value	45.6%	0.0%	30.0%
*AID model estimation results*			
Ln P_Private label carrot rolls_	0.0000	0.0000	0.0000
	(0.0000)	(0.0000)	(0.0000)
Ln P_Brand 1 carrot rolls_	0.0007	0.0000	0.0006
	(0.0006)	(0.0000)	(0.0007)
Ln P_Brand 1 whole-grain rolls_	0.0000	0.0000	0.0000
	(0.0000)	(0.0000)	(0.0000)
Ln P_Brand 2 linseed rolls_	0.0000	0.0000	0.0000
	(0.0000)	(0.0000)	(0.0000)
Real budget	0.0000	0.0000	0.0000
	(0.0000)	(0.0000)	(0.0000)
Reformulation dummy	0.0149	0.0000	0.0151
	(0.0151)	(0.0000)	(0.0166)
Trend	−0.0012	0.0000	−0.0006
	(0.0005)	(0.0000)	(0.0005)
Christmas dummy	0.0001	0.0001	0.0001
	(0.0000)	(0.0000)	(0.0000)
Intercept	0.3657	0.0000	0.2998
	(0.0520)	(0.0001)	(0.0572)
Root MSE	0.0172	0.0000	0.0189
R^2^	0.99	0.98	0.98
R^2^-adj	0.98	0.96	0.96

**Table 9:** AID model estimation results - wholegrain rolls

	Brand 2 sandwich rolls	*Private-label wholegrain rolls*	Brand 2, grain rolls	Brand 1 multi-grain rolls
Initial share of calories	18.1%	35.1%	19.4%	27.4%
Initial share of sales value	16.1%	30.1%	20.5%	33.2%
*AID model estimation results*				
Ln P_Brand 2 sandwich rolls_	0.00003	−0.00001	−0.00001	−0.00001
	(0.00000)	(0.00000)	(0.00000)	(0.00000)
Ln P_Private-label wholegrain rolls_	−0.00001	0.00002	−0.00001	−0.00001
	(0.00000)	(0.00000)	(0.00000)	(0.00000)
Ln P_Brand 2, grain rolls_	−0.00001	−0.00001	0.00002	−0.00001
	(0.00000)	(0.00000)	(0.00000)	(0.00000)
Ln P_Brand 1 multi grain rolls_	−0.00001	−0.00002	−0.00001	0.00004
	(0.00000)	(0.00000)	(0.00000)	(0.00000)
LnP_Brand 3 wholegrain_	−0.00005	0.00008	0.00000	−0.00004
	(0.00006)	(0.00015)	(0.00010)	(0.00011)
Real budget	0.00000	−0.00001	0.00000	0.00001
	(0.00000)	(0.00000)	(0.00000)	(0.00000)
Reformulation dummy	−0.00615	0.00408	0.01379	−0.01164
	(0.00399)	(0.00998)	(0.00683)	(0.00756)
Trend	0.00000	0.00008	−0.00012	0.00005
	(0.00007)	(0.00018)	(0.00012)	(0.00014)
Christmas dummy	−0.00056	0.00234	0.00000	−0.00206
	(0.00534)	(0.01330)	(0.00913)	(0.01010)
AR (1)	0.22398	0.20410	0.38075	0.00000
	(0.02580)	(0.01760)	(0.01950)	(0.00000)
Intercept	0.19093	0.22398	0.20410	0.38075
	(0.01030)	(0.02580)	(0.01760)	(0.01950)
Root MSE	0.0039	0.0098	0.0067	0.0074
R^2^	1.00	0.99	0.99	0.99
R^2^-adj	0.99	0.99	0.99	0.99

**Table 10:** Detailed results - chocolate muesli

	Brand 1	Private label regular	*Private label choco*	Brand 2
Initial share of calories	5.0%	63.8%	21.3%	7.8%
Initial share of sales value	13.1%	46.7%	20.8%	14.4%
*AID model estimation results*				
Ln P_Brand 1_	−0.2943	0.0960	0.0640	0.1247
	(0.0142)	(0.0313)	(0.0177)	(0.0510)
Ln P_Private label regular_	0.6598	−1.2680	−0.6039	0.4600
	(0.4612)	(0.9849)	(0.5757)	(1.5450)
Ln P_Private label choco_	−0.4838	0.5100	0.3138	0.1899
	(0.4055)	(0.8553)	(0.5033)	(1.3312)
Ln P_Brand2_	0.0608	0.2019	0.1427	−0.4529
	(0.0164)	(0.0368)	(0.0205)	(0.0589)
Ln P_Brand 3_	−0.0400	0.1023	0.1085	0.1572
	(0.0692)	(0.1513)	(0.0869)	(0.2413)
Real budget	−0.0257	−0.0966	−0.0236	0.1380
	(0.0128)	(0.0260)	(0.0149)	(0.0413)
Reformulation dummy	−0.0466	0.0570	0.0191	−0.0157
	(0.0334)	(0.0640)	(0.0383)	(0.0992)
Trend	0.0000	0.0002	0.0009	−0.0007
	(0.0008)	(0.0004)	(0.0003)	(0.0006)
Christmas dummy	0.0109	0.0096	−0.0012	0.0165
	(0.0122)	(0.0188)	(0.0126)	(0.0272)
AR (1)	0.8022	0.2210	0.4339	0.0640
	(0.1019)	(0.1694)	(0.1517)	(0.1668)
Intercept	0.7431	2.3112	0.2789	−2.4491
	(0.4261)	(0.9154)	(0.5233)	(1.4814)
Root MSE	0.0148	0.0262	0.0159	0.0413
R^2^	0.94	0.85	0.81	0.89
R^2^-adj	0.92	0.81	0.76	0.86
